# Zeroth Law investigation on the logarithmic thermostat

**DOI:** 10.1038/s41598-018-30129-x

**Published:** 2018-08-03

**Authors:** Puneet Kumar Patra, Baidurya Bhattacharya

**Affiliations:** Department of Civil Engineering, Indian Institute of Technology Kharagpur, Kharagpur, 721302 India

## Abstract

The Zeroth Law implies that the three systems, each separately in equilibrium and having the same temperature, must remain so when brought in pairwise or simultaneous thermal contact with each other. We examine numerically the conformity of the logarithmic thermostat with the Zeroth Law of thermodynamics. Three specific scenarios, with different heat reservoirs, are investigated. For each scenario, the system of interest, *S*_1_ – a single harmonic oscillator, is coupled with two heat reservoirs, *S*_2_ and *S*_3_. *S*_2_ and *S*_3_ are variously chosen to be from the Nosé-Hoover, the Hoover-Holian, the C_1,2_ and the logarithmic thermostats. In the scenarios involving logarithmic thermostat, we observe a violation of the Zeroth Law of thermodynamics, in computationally achievable time, at low to moderate coupling strengths: (i) the kinetic and configurational temperatures of the systems are different, (ii) momentum distribution of log thermostat is non-Gaussian, and (iii) a temperature gradient is created between the kinetic and configurational variables of the log thermostat.

## Introduction

The Zeroth Law of thermodynamics defines an equivalence relation for systems in mutual thermal equilibrium, rendering possible the calibration of thermometers and the measurement of temperature^1,2^. Let the condition of thermal equilibrium between two systems, *S*_1_ and *S*_2_, be given by the unique relation ${F}_{12}({\underline{\theta }}_{1},{\underline{\theta }}_{2})=0$ where ${\underline{\theta }}_{J}$ is the complete set of thermodynamic variables necessary to define the equilibrium state of system *J*(*J* = 1, 2, 3). The Zeroth Law states that^3^

1 $${F}_{12}({\underline{\theta }}_{1},{\underline{\theta }}_{2})=0\,{\rm{and}}\,{F}_{23}({\underline{\theta }}_{2},{\underline{\theta }}_{3})=0\Rightarrow {F}_{13}({\underline{\theta }}_{1},{\underline{\theta }}_{3})=0$$

This transitivity of thermal equilibrium helps establish a common temperature of the three systems and forms the basis of thermometry.

The universality of the Zeroth Law has made it a prerequisite for understanding thermodynamics in the context of both classical and statistical framework. Within the statistical framework, it plays a defining role in deriving the canonical distribution in both the traditional (Gibbs’) treament^4^ as well as the informational theoretic approach^5^. To make the last statement more clear, consider an isolated system comprising of two subsystems, denoted by subscripts 1 and 2. Let the energy of two subsystems be: *E*_1_ and *E*_2_. Let the corresponding entropy be ${{\mathscr{S}}}_{1}$ and ${{\mathscr{S}}}_{1}$. In canonical ensemble, one maximizes the total entropy under the constraint that total energy of the isolated system is constant i.e.:

2 $$\begin{array}{rcl}{{\mathscr{S}}}_{1}+{{\mathscr{S}}}_{2} & = & {\rm{\max }}\,,\\ {E}_{1}+{E}_{2}={E}_{tot} & = & {\rm{constant}}\end{array}$$

While writing these equations, we have assumed that the interaction energy between the two systems is small enough to be neglected. Taking the total differentials of the two^6^ we get:

3 $$\begin{array}{rcl}{{\mathscr{S}}}_{1}^{\text{'}}({E}_{1})d{E}_{1}+{{\mathscr{S}}}_{2}^{\text{'}}({E}_{2})d{E}_{2} & = & 0\\ {E}_{1}^{\text{'}}d{E}_{1}+{E}_{2}^{\text{'}}d{E}_{2} & = & 0\end{array}$$

Dividing the two equations we get ${{\mathscr{S}}}_{1}^{\text{'}}/{E}_{1}^{\text{'}}={{\mathscr{S}}}_{2}^{\text{'}}/{E}_{2}^{\text{'}}=\mathrm{1/}T$. This situation can be generalized to a case where there are *N* subsystems within the system. The fact that the entire system is in equilibrium implies that the subsystems are also in equilibrium with each other. Zeroth law *enables* the partition of the space of thermodynamic states of the subsystems into classes of equivalence. These classes are defined as isotherms, each of which is associated with a unique “empirical temperature”, *T*^7^. Thus, although mutual thermal equilibrium is a prerequisite for the Zeroth Law, the thermodynamic definition of temperature relies on the Zeroth Law of thermodynamics as empirical temperature is defined through it.

In Jaynes’ informational theoretic approach, the Zeroth Law comes into picture through the Lagrangian multiplier associated with the average energy constraint^5,6,8^ and is solved by invoking the argument that the temperature of *S*_1_ is the *same* as *S*_2_ Interested readers are referred to^5^ for more details. It has recently been shown that an initially nonequilibrium system in contact with a heat bath moves, on an average, towards equilibrium, suggesting the validity of the Zeroth (as well as the Second) Law of thermodynamics for thermostatted molecular dynamics^9^.

As is evident from the discussion so far, the Zeroth Law is intrinsically connected to heat reservoirs. An ideal heat reservoir possesses infinite heat capacity so that the energy transferred by the reservoir does not alter its temperature. Such a reservoir is usually assumed to be extremely large. Despite recent advances in computing, we still lack the ability of simulating a system beyond a few billion particles whereas even a mole comprises of 10^23^ particles. In order to get around this limitation, computational models utilize synthetic techniques, called thermostat algorithms, in order to capture the essence of ideal heat reservoirs In simple terms, thermostat algorithms^10–16^, are mathematical constructs to mimic ideal heat reservoirs so that energy exchange processes occurring in real systems may be studied computationally. Regardless of being deterministic or stochastic, the thermostats, when coupled with a system *must* ensure a constant temperature environment for the system as they play the role of a heat-bath^1,17–20^. However, simply ensuring constant temperature computationally does not guarantee that these algorithms *will not violate* established thermodynamic principles. In fact, the merit of a good temperature control algorithm should not only be determined by how well it controls the temperature but also whether it conforms to different thermodynamic and dynamical-systems principles^21^. In this context, the Zeroth Law of thermodynamics is amongst the most fundamental principles of thermodynamics that must be satisfied.

Beginning with conceptually simple velocity rescaling techniques^10^, where velocities are scaled to obtain the desired temperature, we now have several stochastic^11,22^ and deterministic^13–16,23^ algorithms that can sample the dynamics from correct equilibrium distributions while satisfying different thermodynamic properties. These algorithms control either the kinetic temperature^13–15^ defined by,

4 $${k}_{B}{T}_{K}=\frac{\langle \frac{1}{2}\sum _{i=1}^{3N}\frac{{p}_{i}^{2}}{m}\rangle }{3N},$$

or the configurational^22–24^ temperature defined by,

5 $${k}_{B}{T}_{C}=\frac{\langle \nabla {\rm{\Phi }}(\underline{q}\mathrm{).}\nabla {\rm{\Phi }}(\underline{q})\rangle }{\langle \nabla \mathrm{.}\nabla {\rm{\Phi }}(\underline{q})\rangle },$$

or both of them together^16^. Here, Φ(.) denotes the potential energy of the system and ▽ is the gradient operation with respect to the phase-space variables. The control is typically achieved by modifying the Hamiltonian, or equivalently the Newtonian, evolution equations in different ways. However, most of these modifications come with a price – the Hamiltonian formalism is lost. A breakthrough has been provided recently^25,26^ which is the subject of this paper: the logarithmic thermostat with an infinite heat capacity has a Hamiltonian basis. In the present work, we analyze the compatibility of the logarithmic thermostat with the Zeroth Law of thermodynamics.

In the next section, details of the logarithmic thermostat are presented, followed by a brief description of the different non-ergodic and ergodic thermostats employed in the present study. The subsequent sections detail the methodology employed in this study, and the main conclusions drawn from it.

## The logarithmic Thermostat

The logarithmic thermostat, also known as the log oscillator or the log thermostat, is a deterministic thermostat that controls the kinetic temperature, (4), of the system. The name arises due to the logarithmic nature of its Hamiltonian:

6 $${H}_{\mathrm{ln}}=\frac{{p}_{s}^{2}}{2{m}_{s}}+\frac{{k}_{B}{T}_{K}}{2}\,\mathrm{log}(\frac{{s}^{2}+\delta }{b}),$$

where, *s* and *p*_*s*_ denote, respectively, the position and velocity of the thermostat with mass *m*_*s*_, and *b* represents an arbitrary constant with dimensions of length squared (taken as unity in the present study). It is a standard practice to add constant *δ* in the equations for preventing the singularity of the potential energy at origin. Upon invoking the virial theorem under the assumption *δ* ≪ *s*^2^, the following holds true:

7 $$\begin{array}{ccc}\langle {p}_{s}\frac{\partial {H}_{\mathrm{ln}}}{\partial {p}_{s}}\rangle  & = & \langle s\frac{\partial {H}_{\mathrm{ln}}}{\partial s}\rangle \\ \Rightarrow \langle \frac{{p}_{s}^{2}}{m}\rangle  & = & {k}_{B}{T}_{K}\mathrm{.}\end{array}$$

A consequence of (7) is that the kinetic temperature of the thermostat (or in other words, average kinetic energy) is always equal to *k*_*B*_*T*_*K*_, regardless of the total energy of the thermostat. It is also easy to check that the momentum of the logarithmic thermostat is distributed normally^26^. Thus, we see that the logarithmic thermostat can mimic the behavior of an ideal heat reservoir.

Unlike Nosé’s original thermostat^13^, the logarithmic thermostat *theoretically* generates canonical dynamics without necessitating the use of any time scaling parameter. The thermostatted dynamics can be obtained through Hamiltonian equations directly. For example, when a system with Hamiltonian $H={\rm{\Phi }}(\underline{q})+\sum {p}_{i}^{2}\mathrm{/2}m$ is coupled to the logarithmic thermostat through an interaction, *h*(*q*, *s*), the total Hamiltonian of the composite system is given by

8 $${H}_{{\rm{ex}}}={\rm{\Phi }}(\underline{q})+\sum _{i=1}^{3N}\frac{{p}_{i}^{2}}{2m}+\frac{{p}_{s}^{2}}{2{m}_{s}}+\frac{{k}_{B}{T}_{K}}{2}\,\mathrm{log}({s}^{2}+\delta )+h(\underline{q},s\mathrm{).}$$

The resulting equations of motion are:

9 $$\begin{array}{cc}{\dot{q}}_{i}=\frac{{p}_{i}}{m}, & {\dot{p}}_{i}=-\,\frac{\partial {\rm{\Phi }}(\underline{q})}{\partial {q}_{i}}-\frac{\partial h(\underline{q},s)}{\partial {q}_{i}},\\ \dot{s}=\frac{{p}_{s}}{{m}_{s}}, & {\dot{p}}_{s}=-\,\frac{{k}_{B}{T}_{K}s}{{s}^{2}+\delta }-\frac{\partial h(\underline{q},s)}{\partial s},\end{array}$$

and the phase-space distribution of the system is sampled according to:

10 $$\begin{array}{lll}\rho (q,p) & \propto  & \exp (-\beta {H}^{\ast })\\  & = & \exp [-\beta H+\,\mathrm{log}(\frac{\int \exp (-\beta ({H}_{\mathrm{ln}}+h)ds)}{\int \exp (-\,\beta {H}_{\mathrm{ln}}ds)})]\mathrm{.}\end{array}$$

Here, H^*^ is the potential of mean force associated with the system phase-space variables^27^. When the interaction is *weak*, the system follows Gibbs’ distribution $\rho \propto \exp (\beta H)$. In absence of the interaction term, *h*, the system and the logarithmic thermostat may be thought of as separated by an adiabatic wall. The nature of interaction, as we will show later, plays an important role determining the thermodynamic consistency of the logarithmic thermostat. It is important to note that the equations of motion (9) *require* ergodicity in the extended system for a proper sampling from a canonical distribution^26^. While a highly non-linear coupling enhances the ergodicity of the logarithmic thermostat^26^, it comes at the cost of losing Gibbs’ distribution.

However, a logarithmic thermostat cannot be used as a temperature control mechanism in molecular dynamics simulations because of the fundamental deficiencies identified by researchers. The equilibration time, even for small systems, has been estimated to be too large^28^, rendering the numerical implementation unfeasible. Further, the log thermostat does not perform the role of a computational “thermostat” since it does not equilibrate small atomic clusters^29^ and has negative configurational temperature in one dimensional systems. Neither does it allow a heat flow even in presence of a large temperature gradient^30^. Under strong coupling, the log thermostat additionally violates both equipartition and virial theorems^31^.

In the present work, we demonstrate that the logarithmic thermostat violates the Zeroth Law of thermodynamics in computationally achievable time in several scenarios, and relate it to the existing deficiencies highlighted before. Our system of interest, *S*_1_, is a single harmonic oscillator (cf. (1)). In the first scenario, *S*_1_ is coupled with an ergodic heat reservoir, *S*_2_, at *k*_*B*_*T*_*K*/*C*_ = 1 (*T*_*K*/*C*_ denotes controlling either kinetic or configurational temperature). Simultaneously, *S*_1_ is also coupled with an NH thermostatted oscillator, *S*_3_, also kept at *k*_*B*_*T*_*K*_ = 1. In this scenario, the ergodic heat reservoir, *S*_2_, is chosen either as a Hoover-Holian thermostat^21^ (HH) or the higher order configurational thermostat^24^ (*C*_1,2_). As an NH thermostatted oscillator is known to be non-ergodic, it serves as the base test case with which other results are compared. In the second scenario, *S*_3_ becomes a logarithmic thermostat. In the third scenario, both *S*_2_ and *S*_3_ are chosen as logarithmic oscillators.

The three thermostats - NH, HH and *C*_1,2_ are discussed next.

## A nonergodic and two ergodic thermostats

### Nosé-Hoover thermostat

The pioneering work of Nosé^13^ was simplified by Hoover^14^ to give the Nosé-Hoover (NH) equations. NH thermostat revolutionized the field of constant temperature molecular dynamics simulations. It controls the kinetic temperature, (4), by means of a friction-like variable that has its own evolution equation. When coupled with a single harmonic oscillator of unit mass and stiffness at temperature *k*_*B*_*T*_*K*_ = 1, the NH thermostatted equations become:

11 $$\begin{array}{ccc}\dot{q}=p, & \dot{p}=-\,q-\zeta p, & \dot{\zeta }={p}^{2}-1.\end{array}$$

Here, *ζ* represents the effects of the entire heat reservoir. However, the Nosé-Hoover algorithm suffers from the problem of being nonergodic for a single harmonic oscillator^32^. Only 6% of the trajectories are chaotic while the remaining 94% lie on tori^33^.

### Hoover-Holian thermostat

The issue of nonergodicity can be tackled by simultaneously controlling the first two moments of kinetic energy^21^. The resulting Hoover-Holian (HH) thermostat (kept at *k*_*B*_*T* = 1) when coupled with a single harmonic oscillator (with unit mass and stiffness constant) becomes:

12 $$\begin{array}{ll}\dot{q}=p, & \dot{p}=-\,q-\eta p-\xi {p}^{3},\\ \dot{\eta }={p}^{2}-\mathrm{1,} & \dot{\xi }={p}^{4}-3{p}^{2}\mathrm{.}\end{array}$$

Here, *η* and *ξ* denote the thermostat variables that control the first and the second moments of the kinetic energy, respectively. Note that the system is thermostatted at a temperature of unity. Hamiltonian corresponding to the HH equations, (12), remains unknown so far. It is easy to check that the equations of motion represented by (12) satisfy the extended phase-space distribution^33^,

13 $${f}_{{\rm{ex}}}(q,p,\eta ,\xi )\propto {e}^{-\frac{1}{2}[{p}^{2}+{q}^{2}+{\eta }^{2}+{\xi }^{2}]},$$

which is a product of four independent standard normal random variables. The dynamics samples the phase-space in accordance with (13), and unlike the Nosé-Hoover algorithm, results in an ergodic thermostat^21,33^.

### *C*_1,2_ thermostat

The higher-order configurational thermostat (*C*_1,2_ thermostat) is the configurational analogue of the HH thermostat^24^. It controls the first two orders of the configurational temperature using two thermostat variables. The equations of motion of a *C*_1,2_ thermostatted single harmonic oscillator, with unit mass and stiffness, are:

14 $$\begin{array}{ll}\dot{q}=p-\eta q-\xi {p}^{3}, & \dot{p}=-\,q,\\ \dot{\eta }={q}^{2}-\mathrm{1,} & \dot{\xi }={q}^{4}-3{q}^{2}\mathrm{.}\end{array}$$

Here, *η* and *ξ* denote the thermostat variables that now control the first two orders of configurational temperature, respectively. The equations of motion, (14), is able to overcome the nonergodicity of the deterministic first-order configurational temperature based thermostat^23^. The extended phase-space density due to (14) is similar to that shown in (13). It has been shown that, like the HH thermostat, the *C*_1,2_ thermostat has no “holes” in the dynamics, and generates a phase-space distribution that is consistent with the Gibbsian prediction for a single harmonic oscillator.

## Zeroth Law investigations

**Figure 1:**
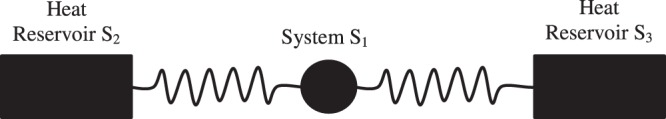
Setup for testing the Zeroth Law. The system, *S*_1_, comprises a single harmonic oscillator. Heat Reservoir *S*_2_ is one of the three thermostats – HH, *C*_1,2_ or the logarithmic thermostat, and the Heat Reservoir *S*_3_ is either the NH thermostatted single harmonic oscillator, or the logarithmic thermostat.

Zeroth Law is concerned with the mutual thermal equilibrium of three bodies. It implies that three systems, each separately in equilibrium and having the same temperature, must remain so when brought in pairwise or simultaneous thermal contact with each other. In the present work, we create a similar scenario (see Fig. 1) – the system of interest, *S*_1_, which is a single harmonic oscillator, is *simultaneously* coupled to two heat reservoirs, *S*_2_ and *S*_3_, both kept at the same temperature. Different scenarios are investigated: in the first scenario, *S*_2_ is one of the two ergodic thermostats (HH or *C*_1,2_) and *S*_3_ is an NH thermostatted oscillator, in the second scenario, *S*_3_ is changed to a logarithmic oscillator while keeping other details the same as in the first scenario, and in the third scenario, both *S*_2_ and *S*_3_ comprise of logarithmic oscillators.The choice of *S*_2_ in the first two scenarios as ergodic is deliberate so that when *S*_1_, the single harmonic oscillator, is coupled to it, equilibration of *S*_1_ occurs according to Gibbsian canonical ensemble. Selecting a non-ergodic thermostat *may* pose problems for thermal equilibration.

For all cases considered here, *S*_1_, the single harmonic oscillator is fully thermalized and has reached an equilibrium state. *S*_1_ is neither subjected to any flux of mass nor energy. The flux of mass may be determined by looking at the average velocity, 〈*p*_1_〉 of the oscillator. Likewise, energy flux may be determined by $\langle {q}_{1}^{2}{p}_{1}+{p}_{1}^{3}\rangle$, where 〈…〉 denotes the time average. For all the different cases investigated in this study, 〈*p*_1_〉 ≈ 0 and $\langle {q}_{1}^{2}{p}_{1}+{p}_{1}^{3}\rangle \approx 0$.

### First Scenario – Zeroth Law for the NH thermostat

In this section, the results of the first scenario are discussed. Two specific cases are considered – (i) Case *A*_1_: *S*_2_ as the HH thermostat, and (ii) Case *A*_2_: *S*_2_ as the *C*_1,2_ thermostat. In both these cases, *S*_3_ is an NH thermostatted harmonic oscillator.

#### Case *A*_1_: *S*_2_ = *HH Thermostat*, *S*_3_ = *NH Thermostat*

The temperature of both heat reservoirs are such that *k*_*B*_*T* = 1. While coupling between the HH thermostat and the single harmonic oscillator is inherent (see (12)), the coupling between the single harmonic oscillator and the NH thermostatted single harmonic oscillator is taken to be harmonic. The combined equations of motion of the system may be written as:

15 $$\begin{array}{ll}{\dot{q}}_{1}={p}_{1}, & {\dot{p}}_{1}=-\,{q}_{1}-\eta {p}_{1}-\xi {p}_{1}^{3}-k({q}_{1}-{q}_{3}),\\ \dot{\eta }={p}_{1}^{2}-\mathrm{1,} & \dot{\xi }={p}_{1}^{4}-3{p}_{1}^{2},\\ {\dot{q}}_{3}={p}_{3}, & {\dot{p}}_{3}=-\,{q}_{3}+k({q}_{1}-{q}_{3})-\zeta {p}_{3},\\ \dot{\zeta }={p}_{3}^{2}-1. & \end{array}$$

Here (*q*_1_, *p*_1_) represent the system variables (*S*_1_), (*η*, *ξ*) represent the HH thermostat (*S*_2_), (*q*_3_, *p*_3_, *ζ*) represent the NH thermostatted oscillator (*S*_3_) and *k* = 0.01, 0.10, 1.00 represents the interaction strength between *S*_1_ and *S*_3_. The equations of motion are solved using classic Runge-Kutta for 100 billion time steps, with each time step being equal to Δ*t* = 0.001. All variables are initialized at unity. Kinetic temperature, *T*_*K*_, of *S*_1_ and *S*_3_ are given by: $\langle {p}_{1}^{2}\rangle$ and $\langle {p}_{3}^{2}\rangle$, respectively, while the configurational temperature, *T*_*C*_, of *S*_1_ and *S*_3_ are given by:

16 $$\begin{array}{lll}{\rm{For}}\,{S}_{1} & : & {T}_{C}=\frac{\langle {({q}_{1}+k({q}_{1}-{q}_{3}))}^{2}\rangle }{1+k}\\ {\rm{For}}\,{S}_{3} & : & {T}_{C}=\frac{\langle {({q}_{3}+k({q}_{3}-{q}_{1}))}^{2}\rangle }{1+k}\end{array}$$

Since both reservoirs are kept at the *same* temperature, given sufficient time, *T*_*K*_ of *S*_1_, *S*_2_ and *S*_3_ must agree with each other according to the Zeroth Law, and so must *T*_*C*_. Not only that, being in equilibrium necessarily means that *T*_*K*_ and *T*_*C*_ must be the same for each system. All these equalities are demonstrated in Table 1, the maximum difference from the desired values being smaller than 0.6%. Later on, we will see that these essential features are not retained when *S*_3_ is replaced by a logarithmic thermostat.

**Table 1:** Time averaged value of kinetic and configurational temperatures, T_*K*_ and T_*C*_, respectively, for the various cases investigated in this study.

*k*	Case *A*_1_				Case *A*_2_				Case *B*_1_				Case *B*_2_				Case *C*_1_	
	*T*_*K*_		*T*_*C*_		*T*_*K*_		*T*_*C*_		*T*_*K*_		*T*_*C*_		*T*_*K*_		*T*_*C*_		*T*_*K*_	
	*S*_1_	*S*_3_	*S*_1_	*S*_3_	*S*_1_	*S*_3_	*S*_1_	*S*_3_	*S*_1_	*S*_3_	*S*_1_	*S*_3_	*S*_1_	*S*_3_	*S*_1_	*S*_3_	*S*_1_	S3
0.0	—	—	—	—	—	—	—	—	—	—	—	—	—	—	—	—	1.003	1.000
0.01	0.997	1.002	1.006	1.004	1.001	1.002	1.004	1.011	1.000	1.047	1.000	2.726	0.999	0.944	1.000	1.052	1.979	1.986
0.1	1.003	1.000	1.001	1.000	1.001	0.997	0.998	1.001	0.999	1.020	1.000	1.284	1.001	0.966	1.000	0.986	2.000	1.996
1.0	1.001	1.001	1.005	1.000	0.999	1.000	1.000	0.999	1.001	0.999	0.999	0.993	1.000	1.000	1.002	0.999	2.009	2.010

The desired temperature is unity. Notice, the difference between *T*_*K*_ and *T*_*C*_ for cases *B*_1_ and *B*_2_ that involve log thermostat as *S*_3_. *S*_1_, the single harmonic oscillator, displays correct temperature. In these cases, a temperature gradient is not only created between *S*_1_ and *S*_3_, but also within the configurational and kinetic variables of *S*_3_. For case *C*_1_, where *S*_2_ and *S*_3_ are log thermostats, the instant a coupling is introduced, the temperature of the system goes haywire. Please note that the temperature corresponding to *S*_2_ were found to be statistically indifferent from that of *S*_1_ (except in case *C*_1_), and hence not listed for the first two cases.

**Figure 2:**
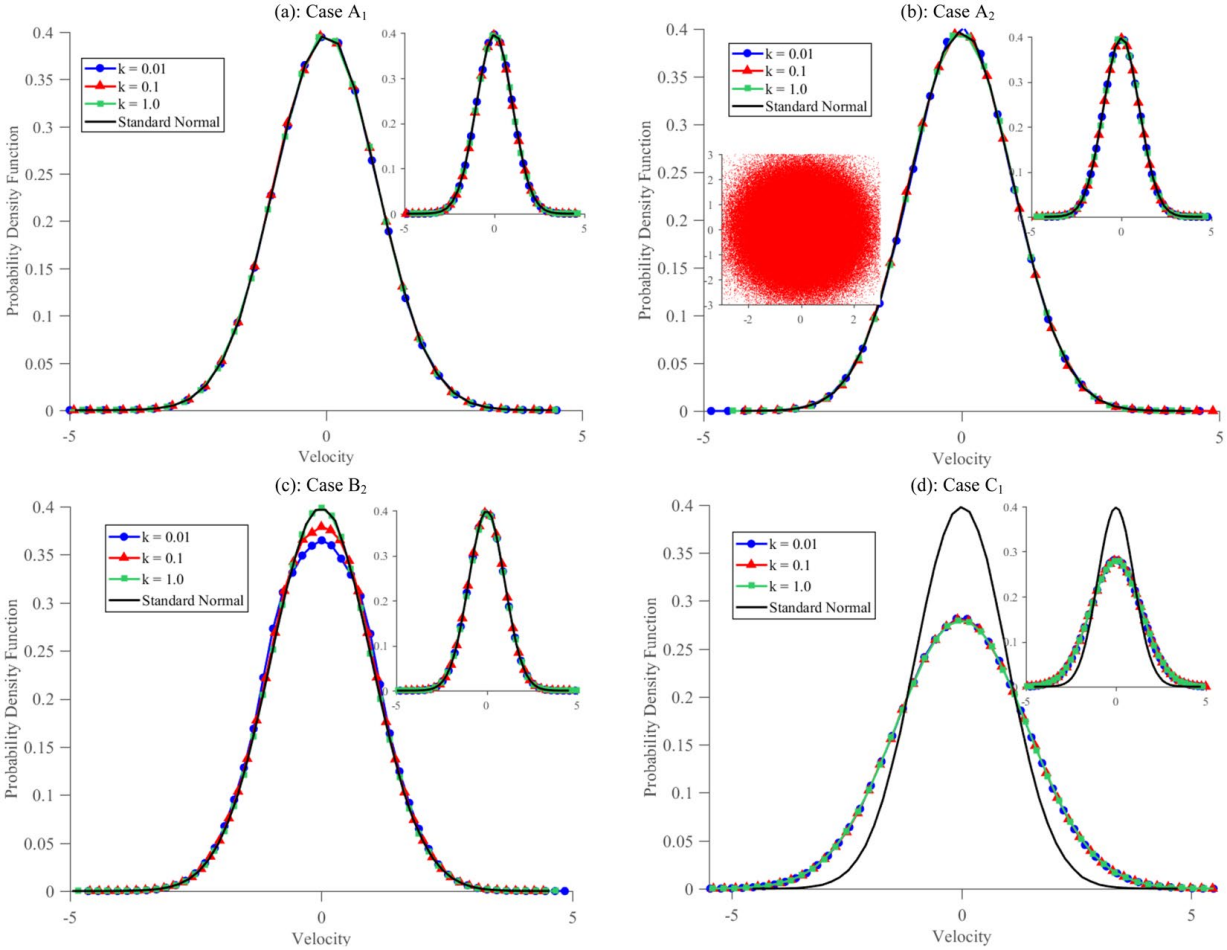
Momentum distributions of *S*_3_ and *S*_1_ (right inset) for the different cases analyzed in the work: (**a**) Case *A*_1_ with *S*_2_ = HH thermostat and *S*_3_ = NH thermostat, (**b**) Case *A*_2_ with *S*_2_ = *C*_1,2_ thermostat, *S*_3_ = NH thermostat, (**c**) Case *B*_1_ with *S*_2_ = HH thermostat and *S*_3_ = Log thermostat, and (**d**) Case *C*_1_ with both *S*_2_ and *S*_3_ = Log thermostat. For each case, *S*_1_ is a single harmonic oscillator. Cases with *S*_3_ = NH thermostat have the correct standard normal distribution of momentum irrespective of the system. For Case *B*_1_, correct momentum distribution of *S*_1_ is obtained at all coupling strengths, however, *S*_3_ has the correct momentum distribution only at high coupling. For case *C*_1_, the computed temperature is double that of desired temperature. Conformity of the velocity distributions with each other and with a standard normal distribution suggests that the Zeroth Law is satisfied only for cases *A*_1_ and *A*_2_.

An additional consequence of the Zeroth Law is the canonical nature of the momentum distribution function for each of *S*_1_, *S*_2_ and *S*_3_, which in this case implies a standard normal distribution. Such a distribution is possible for *S*_3_ only when the NH thermostatted oscillator displays ergodicity. The marginal momentum distributions, shown in Fig. 2(a), are in agreement with the standard normal distribution irrespective of coupling strength. Note that a more complete proof of canonical nature involves looking at joint probability distribution functions^34^. Other ergodic oscillators, when coupled with the HH oscillator also show similar features^33^. As would be seen later, such conformity is typically absent for the logarithmic thermostat at low to moderate coupling interaction (see Fig. 2(c)). A failure to demonstrate the correct momentum distribution would have indicated a deviation from canonical nature, which in turn would have implied a lack of equilibrium, and hence would have violated the Zeroth Law.

#### Case *A*_2_ : *S*_2_ = *C*_1,2_*Thermostat*, *S*_3_ = *NH Thermostat*

This case presents an interesting situation – the *C*_1,2_ thermostat controls *only* the configurational temperature by acting upon the configurational variables, while the NH thermostat controls *only* the kinetic temperature by altering the momentum evolution equations. Equality of configurational (as well as kinetic) temperatures throughout the composite system provides a mechanism for checking if the Zeroth Law holds true in this case. The equations of motion solved in this case are:

17 $$\begin{array}{ll}{\dot{q}}_{1}={p}_{1}+\eta F+\xi UF, & {\dot{p}}_{1}=F,\\ \dot{\eta }={F}^{2}-\mathrm{(1}+k), & \dot{\xi }=U{F}^{2}-[U(1+k)+{F}^{2}],\\ {\dot{q}}_{3}={p}_{3}, & {\dot{p}}_{3}=-\,{q}_{3}+k({q}_{1}-{q}_{3})-\zeta {p}_{3},\\ \dot{\zeta }={p}_{3}^{2}-\mathrm{1,} & \end{array}$$

where, *F* = −[*q*_1_ + *k*(*q*_1_ − *q*_3_)] and *U* = *k*(*q*_1_ − *q*_3_)^2^. The expressions of *T*_*K*_ and *T*_*C*_ for both *S*_1_ and *S*_3_ remain the same as in case *A*_1_. For *T*_*C*_ to be equal for *S*_1_ and *S*_3_, the following must hold true:

18 $$\begin{array}{rcl}\langle {({q}_{3}+k({q}_{3}-{q}_{1}))}^{2}\rangle  & = & \langle {({q}_{1}+k({q}_{1}-{q}_{3}))}^{2}\rangle \\ \Rightarrow \langle {q}_{3}^{2}\rangle  & = & \langle {q}_{1}^{2}\rangle \mathrm{.}\end{array}$$

It is easy to check that for the composite system (assuming ergodicity), 〈*q*_3_〉 = 〈*q*_1_〉 = 0. Thus, in this case, apart from the equality of kinetic and configurational temperatures, we perform additional tests on the equality of the first and the second moments of the variables *q*_1_ and *q*_3_. The equations of motion are solved using classic Runge-Kutta for 100 billion time steps, with each time step being equal to Δ*t* = 0.001.

The results for this case, shown in Table 1, are found to be essentially the same as that of case *A*_1_: (i) *T*_*K*_ of *S*_1_ and *S*_3_ agree with each other, (ii) *T*_*C*_ of *S*_1_ and *S*_3_ agree with each other, and (iii) *T*_*K*_ and *T*_*C*_ of each system agree with each other. $\langle {q}_{1}^{2}\rangle$ and $\langle {q}_{3}^{2}\rangle$ are found to be equal as are 〈*q*_1_〉 = 〈*q*_3_〉 = 0 are demonstrated numerically in Table 2. The marginal momentum distribution functions for *S*_3_ and *S*_1_ are shown in Fig. 2(b) and its right inset, respectively. Both the NH oscillator as well as *S*_1_ show a remarkable conformity with the standard normal distribution, just like in case *A*_1_. The inset on the left shows the phase space plot of the NH oscillator, (*q*_3_, *p*_3_), highlighting its ergodic nature.

**Table 2:** Case *A*_2_: Verification of 〈*q*_1_〉 = 〈*q*_3_〉 = 0 and $\langle {q}_{1}^{2}\rangle =\langle {q}_{3}^{2}\rangle$ which is a consequence of ergodicity.

*k*	〈*q*_1_〉	〈*q*_3_〉	$\langle {{\boldsymbol{q}}}_{{\bf{1}}}^{{\bf{2}}}\rangle$	$\langle {{\boldsymbol{q}}}_{{\bf{3}}}^{{\bf{2}}}\rangle$
0.01	−0.001	0.002	0.995	1.006
0.1	0.001	0.000	0.959	0.958
1.0	−0.001	0.000	0.816	0.815

### Second Scenario – Zeroth Law for the logarithmic thermostat

We now investigate what happens when NH thermostat of the first scenario is replaced by a logarithmic thermostat (cases *B*_1_ and *B*_2_). The equations of motion with a log-thermostat are “stiff”, and require smaller time-step for numerical integration. As a result, an integration time-step of Δ*t* = 0.00025 is used. The equations of motion are solved for 800 billion time steps with classic 4^*th*^ order Runge-Kutta algorithm. All variables are initialized at unity, unless otherwise specified.

#### *Case B*_1_: *S*_2_ = *HH Thermostat*, *S*_3_ = *Log Thermostat*

In this case, the single harmonic oscillator (*S*_1_) is coupled with the ergodic HH thermostat (*S*_2_) and the logarithmic thermostat (*S*_3_). The coupling between *S*_1_ and *S*_3_ is taken as harmonic, with spring constant *k*. The equations of motion are:

19 $$\begin{array}{ll}{\dot{q}}_{1}={p}_{1}, & {\dot{p}}_{1}=-\,{q}_{1}-\eta {p}_{1}-\xi {p}_{1}^{3}-k({q}_{1}-{q}_{3}),\\ \dot{\eta }={p}_{1}^{2}-\mathrm{1,} & \dot{\xi }={p}_{1}^{4}-3{p}_{1}^{2},\\ {\dot{q}}_{3}={p}_{3}, & {\dot{p}}_{3}=-\,\frac{{q}_{3}}{{q}_{3}^{2}+\delta }+k({q}_{1}-{q}_{3})\end{array}$$

Variables *q*_3_ and *p*_3_ denote the logarithmic thermostat’s position and momentum, respectively. We keep *δ* = 0.01, and consider three values of the spring constant *k* = 0.01, 0.1 and 1.0, denoting, respectively, the cases of weak, moderate and strong interaction with the system. *T*_*K*_ for *S*_1_ and *S*_3_ are: $\langle {p}_{1}^{2}\rangle$ and $\langle {p}_{3}^{2}\rangle$, respectively. The expressions for *T*_*C*_ for *S*_1_ and *S*_3_ are:

20 $$\begin{array}{lll}{\rm{For}}\,{S}_{1} & : & {T}_{C}=\frac{\langle {({q}_{1}+k({q}_{1}-{q}_{3}))}^{2}\rangle }{1+k}\\ {\rm{For}}\,{S}_{3} & : & {T}_{C}=\frac{\langle {(\frac{{q}_{3}}{{q}_{3}^{2}+\delta }+k({q}_{3}-{q}_{1}))}^{2}\rangle }{\langle \frac{\delta -{q}_{3}^{2}}{{({q}_{3}^{2}+\delta )}^{2}}+k\rangle }\end{array}$$

**Figure 3:**
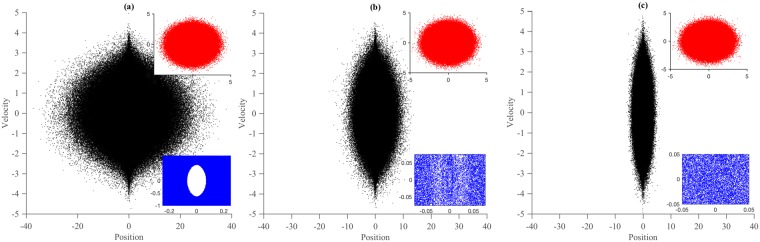
Phase space trajectory of *S*_3_ in case *B*_1_ due to: (**a**) weak interaction at *k* = 0.01, (**b**) moderate interaction at *k* = 0.10 and strong interaction at *k* = 1.0. The inset figures at top (in red) represent the phase-space trajectory of the *S*_1_ at the corresponding interaction strengths. The inset figures at bottom (in blue) a provide a zoomed-in view of the trajectory of *S*_3_. Notice the difference in the phase-space trajectories between the logarithmic thermostat and the single harmonic oscillator at a particular interaction strength. The zoomed-in inset views show a hole in the dynamics at weak interaction and improper sampling around origin at moderate interaction.

The existence of a single unique temperature of a system is necessary for the Zeroth Law of thermodynamics to hold true^35^. Further the different measures of temperature are necessarily equal for a closed equilibrium system^7,36,37^. In fact, extending the Zeroth Law for non-equilibrium situations is problematic because of the absence of a unique value of temperature^38,39^ of a system. *T*_*K*_ and *T*_*C*_ of the different oscillators for this case are shown in Table 1. Unlike in the first scenario, here we observe that at low to moderate coupling strengths, *T*_*K*_ of the logarithmic thermostat does not reach the desired value of unity during the simulation run – a deviation of 2% to 5% is observed, which is significant compared to the cases *A*_1_ and *A*_2_. *T*_*C*_, on the other hand, deviates from the desired value even more – 28% to 172%. Further, *T*_*C*_ ≠ *T*_*K*_ for the logarithmic thermostat – a clear violation of the Zeroth Law. Interestingly, the single harmonic oscillator, *S*_1_, faithfully reproduces the desired kinetic and configurational temperatures. Further, at weak and moderate interaction strengths, the dynamics of the logarithmic thermostat is substantially different from that of the single harmonic oscillator (see Fig. 3). Although the dynamics of the logarithmic thermostat appears to be phase-space filling, a majority of the trajectory points are confined within a small region. This problem is predominant at small and moderate interaction strengths. In fact, for *k* = 0.01, there is an evidence of a hole in the dynamics.

The information embedded in the momentum distribution functions is more detailed than just its second moment. In canonical ensemble, in addition to the standard deviation of momentum distribution being equal to the temperature, the entire distribution must also be Gaussian. Utilizing this, in the previous scenario, we argued that the NH thermostatted oscillator displays a good thermalizing behavior. However, in the present case, the velocity distribution function, shown in Fig. 2(c), shows a marked deviation from Gaussianity at low and moderate coupling. In other words, the phase-space of the logarithmic thermostat does not get sampled from a canonical ensemble as evidenced from Fig. 3. At strong interaction, however, the velocity distribution improves, and the deviation ceases to exist, but this comes at a cost: the dynamics now samples from (10) instead of the standard canonical distribution function. The single harmonic oscillator, on the other hand, always demonstrates faithfully a Gaussian velocity distribution. The improved behavior of the log thermostat at strong coupling makes us conjecture that instead of *S*_3_ thermalizing *S*_1_, it is the other way around.

Thus, in this case we observe that, in computationally achievable time, – (i) there are significant differences between the temperatures (both *T*_*K*_ and *T*_*C*_) of *S*_1_ and *S*_3_ in equilibrium, (ii) a temperature difference is created between the momentum and the configurational variables of *S*_3_, violating the principles of equilibrium thermodynamics, (iii) at low to moderate coupling, the phase-space of *S*_3_ does not get sampled from a canonical distribution, rendering the momentum distributions different from a standard normal distribution.

#### *Case B*_2_: *S*_2_ = *C*_1,2_*Thermostat*, *S*_3_ = *Log Thermostat*

In this case, the HH thermostat is replaced with the *C*_1,2_ thermostat as *S*_2_. The equations of motion to be solved are:

21 $$\begin{array}{ll}{\dot{q}}_{1}={p}_{1}+\eta F+\xi UF, & {\dot{p}}_{1}=F,\\ \dot{\eta }={F}^{2}-\mathrm{(1}+k), & \dot{\xi }=U{F}^{2}-[U(1+k)+{F}^{2}],\\ {\dot{q}}_{3}={p}_{3}, & {\dot{p}}_{3}=-\,\frac{{q}_{3}}{{q}_{3}^{2}+\delta }+k({q}_{1}-{q}_{3}),\end{array}$$

where, *F* = −[*q*_1_ + *k*(*q*_1_ − *q*_3_)] and *U* = *k*(*q*_1_ − *q*_3_)^2^. The expressions of *T*_*K*_ and *T*_*C*_ remain the same as in case *B*_1_.

Time averaged values of *T*_*K*_ and *T*_*C*_ of *S*_1_ and *S*_3_, for this case, are shown in the Table 1. Like in case *B*_1_, the situation does not improve here at low and moderate interaction strengths. While *S*_1_ again faithfully demonstrates the correct kinetic and configurational temperatures, such is not the case for the logarithmic thermostat. The inequality of *T*_*K*_ and *T*_*C*_ for the logarithmic thermostat, at low and moderate interaction strengths, suggest a non-unique temperature of the system, and effectively creates a temperature gradient between the kinetic and configurational variables, unlike in case *A*_2_. Thus, again a violation of the Zeroth Law, in computationally achievable time, is observed.

### Third Scenario – Zeroth Law with two coupled logarithmic thermostats

#### *Case C*_1_: *S*_2_ = *Log Thermostat*, *S*_3_ = *Log Thermostat*

**Figure 4:**
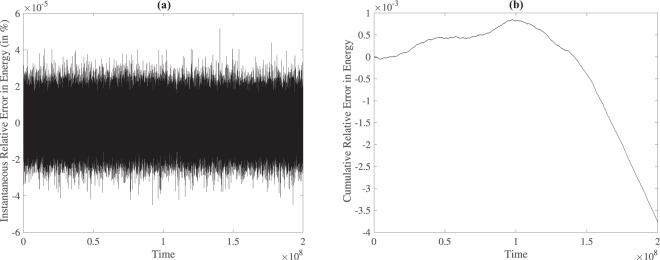
(**a**) Instantaneous Relative Error in Energy and (**b**) Cumulative Relative Error in Energy for case *C*_1_ with *k* = 0.01.

We now investigate the third scenario where two logarithmic thermostats are coupled to *S*_1_ harmonically, but with different strengths, *k* and k*. A similar situation was investigated before in nonequilibrium^30^ – *S*_1_ comprised of a *ϕ*^4^ chain, and a temperature difference was created between the two ends of the chain through two logarithmic thermostats. However, no heat flow was observed. In the present scenario, the temperatures of the two thermostats are kept at unity. The harmonic coupling between the thermostats is taken such that the evolution equations are:

22 $$\begin{array}{rcl}{\dot{q}}_{2} & = & {p}_{2}\\ {\dot{p}}_{2} & = & -\,\frac{{q}_{2}}{{q}_{2}^{2}+\delta }+{k}^{\ast }({q}_{1}-{q}_{2})\\ {\dot{q}}_{1} & = & {p}_{1}\\ {\dot{p}}_{1} & = & -{k}^{\ast }({q}_{1}-{q}_{2})-k({q}_{1}-{q}_{3})\\ {\dot{q}}_{3} & = & {p}_{3}\\ {\dot{p}}_{3} & = & -\frac{{q}_{3}}{{q}_{3}^{2}+\delta }+k({q}_{1}-{q}_{3})\end{array}$$

k* is chosen as 1.0, while three values of *k* are used: 0.01, 0.1, 1.0 with *δ* equaling 0.01. The initial conditions are taken as: (*q*_2_, *p*_2_, *q*_1_, *p*_1_, *q*_3_, *p*_3_) = (1, 2, 2, 3, 3, 4). This third scenario corresponds to a situation where one can define a Hamiltonian. However, we still employ the non-symplectic 4^*th*^ order Runge-Kutta method for solving the equations of motion to maintain consistency. The fluctuations in total energy of the system is of the order of 10^−7^, the relative error (in %) is of the order of 10^−5^, and the cumulative error is of the order of 10^−3^ as shown in Fig. 4. Since our objective is not to study the energy conserving nature of the log thermostat, using the 4^*th*^ order Runge-Kutta method for solution does not have any significant bearing. We remind the readers that the equations of motion (22) correspond to the case where the temperature is set at unity. As a consequence, the velocities of the logarithmic thermostats for all cases *must* sample from a standard-normal distribution. The velocity distributions, which are both non-gaussian, are shown in Fig. 2(d).

Despite 800 billion integration time steps, at small values of $k > 0$, we observe *T*_*K*_ of the two oscillators to be different (see Table 1). While the Zeroth Law is satisfied for the moderate and strong interaction, it is disconcerting to see that *T*_*K*_ is twice the desired temperature in every case. Note that *T*_*K*_ has been computed as ${\langle {p}_{i}^{2}\rangle }_{t}$, the time-averaged value of second moment of velocity. The results are around $\sqrt{2}$ instead, if the temperatures were computed as 〈(*p*_*i*_ − *E*[*p*_*i*_])^2^〉_*t*_, the second moment of velocity around its mean. Interestingly, when *k* = 0, i.e. *S*_3_ is decoupled, the log-thermostats behave expectedly, with temperature commensurate with the desired temperature of unity, and the values are independent of the nature of second moment (central or non-central). It has been previously argued that the details of the thermal contact are not important^40^, however, we find system temperature to change with changing values of *k*.

## Summary and Conclusions

Zeroth Law helps us to identify the temperature of a statistical-mechanical system, and forms a cornerstone of thermodynamics. Recently, it has been shown mathematically that a non-isothermal system relaxes to canonical equilibrium conditions, with all components of the system having the same temperature^41^. Therefore, if two thermostatted systems (at same temperature) are coupled to each other, each of them *must* individually satisfy the Zeroth Law. In this article, we explore numerically if the Zeroth Law is satisfied for the logarithmic thermostat. The summary of findings are shown in Table 3.

**Table 3:** Summary of findings. SHO = single harmonic oscillator.

Case	*S*_1_	*S*_2_	*S*_3_	Findings
*A*_1_	SHO	HH thermostat	NH thermostat	For all coupling strengths in case A:
*A*_2_		*C*_1,2_ thermostat		1. *T*_*K*_ and *T*_*C*_ of *S*_1_, *S*_2_ and *S*_3_ are in agreement with each other.
				2. Momentum distribution follows a standard normal distribution
*B*_1_	SHO	HH thermostat	Log thermostat	At low to moderate coupling strengths:
				1. *T*_*K*_ and *T*_*C*_ of *S*_1_, *S*_2_ and *S*_3_ are different from each other
*B*_2_		*C*_1,2_ thermostat		2. Momentum distribution of *S*_3_ is not standard normal.
				At larger coupling strengths, Gibbs’ canonical sampling is lost.
*C*_1_	SHO	Log thermostat	Log thermostat	Momentum distribution is not standard normal and temperature control fails

**Figure 5:**
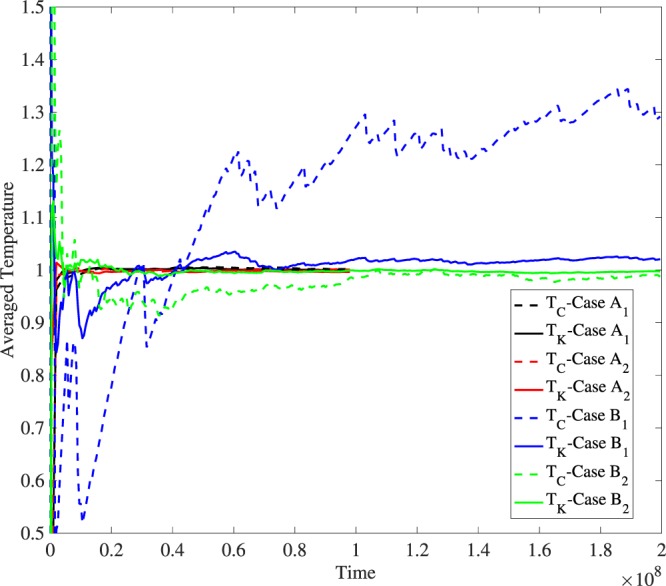
Time evolution of averaged temperatures for *S*_3_ under different cases. Note that in cases *A*_1_ and *A*_2_, i.e. with the NH thermostat as *S*_3_, convergence to the desired value of unity is achieved very quickly. On the other hand, for cases *B*_1_ and *B*_2_, such convergence is typically absent throughout. The results are plotted with *k* = 0.1.

The temporal evolution of *T*_*K*_ and *T*_*C*_ for *S*_3_ in cases *A*_1_, *A*_2_, *B*_1_ and *B*_1_ for *k* = 0.1 are shown in Fig. 5. Note that in cases *A*_1_ and *A*_2_, i.e. with the NH thermostat as *S*_3_, convergence to the desired value of unity is achieved very quickly. On the other hand, for cases *B*_1_ and *B*_2_, such convergence is typically absent throughout. The picture does not change with *k* = 0.01. Our results indicate that coupling an ergodic system with the logarithmic thermostat does not guarantee a canonical distribution for the logarithmic thermostat at small to moderate interaction strengths, and consequently it may display an incorrect temperature. When two logarithmic thermostats are coupled, the combined system goes haywire – the temperature of all components shoot up to twice the desired value. kinetic temperature of both the logarithmic thermostats is almost twice the desired value.

Violation of the Zeroth Law by the logarithmic thermostat *in computationally achievable time* is a consequence of the flaws demonstrated previously by other researchers^28–31^. In view of the large equilibration time of the log thermostat^28^ and its inability to engender heat flow^30^, the heat flow within the single harmonic oscillator is approximately zero despite the differences in *T*_*K*_ and *T*_*C*_ of the single harmonic oscillator and the logarithmic thermostat. Coupling to a “good” thermostatted system improves the phase-space sampling of the logarithmic thermostat in some cases, however, the improvement is not sufficient to make its *T*_*K*_ = *T*_*C*_ primarily because of its poor thermalizing behavior^29^. At strong coupling, we find that the improvement in the performance comes at the cost of violating the equipartition and virial theorems^31^.

Lastly, the method outlined in this paper may serve as a test for the goodness of other thermostats as well.
